# Patients with multimorbidity and their experiences with the healthcare process: a scoping review

**DOI:** 10.15256/joc.2017.7.97

**Published:** 2017-01-27

**Authors:** Maartje J. van der Aa, Jennifer R. van den Broeke, Karien Stronks, Thomas Plochg

**Affiliations:** ^1^Department of Health Services Research, Maastricht University, Maastricht, The Netherlands; ^2^Department of Public Health, Academic Medical Center, University of Amsterdam, The Netherlands

**Keywords:** Multimorbidity, delivery of healthcare, patient experience, professional-patient relation, healthcare system, scoping review

## Abstract

**Background:**

The number of patients with multimorbidity (two or more conditions) is increasing. Observational research has shown that having multiple health problems is associated with poorer outcomes in terms of health, quality of care, and costs. Thus, it is imperative to understand how patients with multimorbidity experience their healthcare process. Insight into patient experiences can be used to tailor healthcare provision specifically to the needs of patients with multimorbidity.

**Objective:**

To synthesize self-reported experiences with the healthcare process of patients with multimorbidity, and identify overarching themes.

**Design:**

A scoping literature review that evaluates both qualitative and quantitative studies published in PubMed, Embase, MEDLINE, and PsycINFO. No restrictions were applied to healthcare setting or year of publication. Studies were included if they reported experiences with the healthcare process of patients with multimorbidity. Patient experiences were extracted and subjected to thematic analysis (interpretative), which revealed overarching themes by mapping their interrelatedness.

**Results:**

Overall, 22 empirical studies reported experiences of patients with multimorbidity. Thematic analysis identified 12 themes within these studies. The key overarching theme was the experience of a lack of holistic care. Patients also experienced insufficient guidance from healthcare providers. Patients also perceived system-related issues such as problems stemming from poor professional-to-professional communication.

**Conclusions:**

Patients with multimorbidity experience a range of system- and professional-related issues with healthcare delivery. This overview illustrates the diversity of aspects that should be considered in designing healthcare services for patients with multimorbidity.

## Introduction

The number of patients with multimorbidity is increasing; representing 50% of the disease burden in most member states of the Organisation for Economic Co-operation and Development (OECD) [1–3]. Patients with multimorbidity are a heterogeneous group with varying diagnoses and number of conditions; however, the impact of multimorbidity on the healthcare process and outcomes is becoming increasingly clear. Observational research indicates that having multiple health problems is associated with poorer outcomes in terms of longer hospital stays [4], more avoidable admissions, and complications [5]. Moreover, associations between the number of chronic conditions and both increased service utilization and higher cost has been identified [6–8]. It is of interest to explore whether the poorer outcomes of care are reflected in self-reported experiences of patients with multimorbidity with the healthcare process. Some evidence supports this hypothesis; for example, a secondary data analysis has shown that complaints are more likely to be lodged by patients with multimorbidity than by other patients with single diseases [9].

In light of this evidence, it seems both timely and relevant to synthesize the evidence regarding the healthcare experiences of patients with multimorbidity. A better understanding of these experiences would help to adapt healthcare to the needs of the patient and thereby improve their healthcare. Different empirical studies report patient experiences in specific settings. However, there is no coherent understanding of the overarching themes from the perspective of patients with multimorbidity [10]. As a result, efforts to improve care for patients with multimorbidity are mostly based on context-specific data and/or one particular aspect of healthcare delivery. For example, it is known that there is a mismatch between the interdepartmental needs of patients with multimorbidity and the departmental organizational structure, which leads to experienced problems in care coordination [e.g. 11,12]. This particular aspect is conceptualized as an organizational problem and accordingly attended to by organizational solutions such as integrated healthcare [13]. However, it is known that patients with multimorbidity encounter problems beyond coordination aspects. Specific patient experiences may point towards the patient perspective; however a coherent overview is warranted to inform the development of multimorbidity care. It is acknowledged that such overarching evidence is vital in the design of evidence-based healthcare in general, and especially to address the needs of the increasing number of patients with multiple conditions [10]. Therefore, this study retrospectively analyses and provides a coherent overview of the body of evidence on the experiences of patients suffering from multiple conditions.

## Objectives

To synthesize the experiences of patients with multimorbidity within the healthcare system into a comprehensive overview and to identify overarching themes underlying these experiences. Such an understanding of the patient perspective might facilitate better adaptation of healthcare services to the specific needs of the increasing number of patients with multiple conditions.

## Methods

A scoping literature review aims to gain a broad understanding of a research area, and more specifically, to gain an overview of various research findings [14]. A scoping review includes different types of evidence to improve coverage of all relevant topics. The methodology involves a comprehensive, non-systematic search of the literature, but does not necessarily identify all available sources. For the current study, both qualitative and quantitative evidence, which were synthesized by data-driven (inductive) thematic analysis, were utilized. The study consisted of three stages: study selection, data extraction, and data analysis.

### Study selection

The following databases for health services research were searched up until May 11, 2016: PubMed and Ovid (Embase, MEDLINE, and PsycINFO). The search was not restricted by publication date or geographic location. However, a filter was applied for language (English or Dutch). We used the following search strategy: (“comorbidity” OR “multimorbidity”) AND (“patient perspective” OR “patient experience” OR “patient satisfaction”).

The first step of the selection process involved reading the titles of all retrieved sources. Studies were selected if the title mentioned either multiple conditions (comorbidity or multimorbidity) or a patient perspective. Two reviewers (M.A. and J.B.) independently selected the studies: a study selected by either reviewer was included for the initial assessment. Abstracts of selected sources were then assessed similarly by both reviewers based on three criteria, which all had to be met for inclusion: (1) the investigated population had to include patients with two or more conditions; (2) the outcome measures had to include self-reported patient experiences, i.e. beyond satisfaction, with the healthcare process; and (3) evidence had to be empirical (either qualitative or quantitative). Finally, the same criteria were applied when processing the full-text articles of the remaining selection. The two reviewers independently assessed the studies to determine whether they were relevant to addressing the research question. If the reviewers disagreed at this stage, consensus was reached by discussion.

### Data extraction

Self-reported experiences with the healthcare process were extracted from the selected articles (M.A. with a check by J.B.). For the qualitative studies, extracted experiences were either actual statements expressed by the patients or a description of their experiences as presented by the authors of the article. For the quantitative studies, extracted experiences consisted of the measures employed in the questionnaires of those papers, indicating patients’ perceptions. Experiences did not have to be related exclusively to multimorbidity; experiences perceived in cases of single diseases were also extracted because they had been mentioned and were, therefore, also considered to be part of the perspective of patients with multimorbidity.

Articles also reported patients’ general reflections and preferences regarding the healthcare process. Although these are not actual experiences, one can derive patients’ problems from their expectations when they indicate a gap between the actual and desired situation - an implicitly expressed experience. For example, a respondent’s reflection that “doctors should attend to the unique needs of patients” implies that a patient experiences that doctors do not attend to their unique needs.

### Data analysis

We constructed an overview of the experiences of patients with multimorbidity by synthesizing different types of evidence using data-driven thematic analysis [15]. This analysis is not a standard technique and allows considerable flexibility for integrating different types of evidence [16]. All experiences were extracted and both quantitative and qualitative evidence was subjected to an open coding process. This was performed by assigning codes to extracted evidence, statement by statement. Statements often included more than one experience and were connected to each other in the perception of the patient. These statements were assigned more than one code and recorded as a unity of parts. These multi-coded statements, therefore, point at interrelatedness of experiences as reported by these patients.

All codes were grouped into separate concepts. The concepts were then organized to create another level of more abstract categories. This was done in two phases. First, three qualitative articles – that contained high numbers of experiences [17–19] – were analysed independently by two researchers (M.A. and J.B.). In the event of a disagreement, consensus was reached by discussion. Further coding and grouping was performed by one researcher (M.A.), with each step being checked and agreed upon by a second researcher (J.B.).

Finally, relationships between categories of experiences were mapped by analysing the statements of patients that were assigned more than one code due to interrelatedness. Patients’ self-reported links were re-established by translating the relationships identified at the coding level into relationships at the abstract category level. This provides insight into the interrelatedness of categories in the healthcare process. To arrive at a concise overview of the perspective of patients with multimorbidity, a map was constructed from the relationships between different experiences (category level) that were mentioned by at least three of the source articles.

## Results

**Figure 1:**
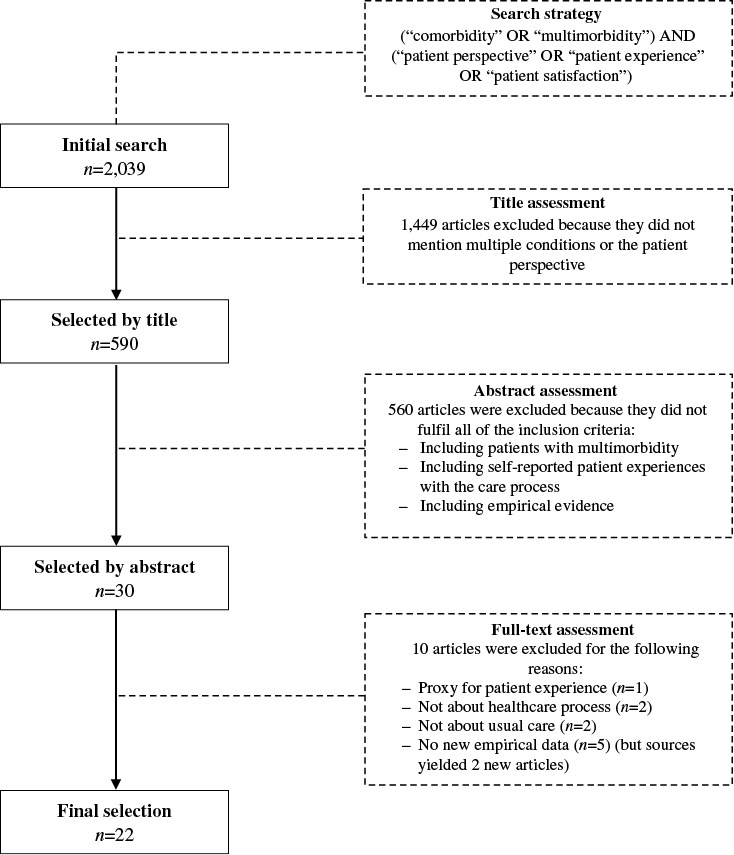
Flowchart of study selection.

The search resulted in 2,039 unique records. Title selection excluded 1,449 studies because neither patient experiences nor multiple conditions (comorbidity or multimorbidity) were mentioned. Abstracts of the remaining 590 articles were assessed, and 30 articles fulfilled all of the inclusion criteria. Based on full-text assessment, 10 studies were excluded for the following reasons: proxy for patient experiences (1), no experiences with the healthcare process (2), no usual care (2), or no new empirical data (5). However, the latter group contained two review articles, which were assessed for further sources of evidence. Two additional articles were identified that met the selection criteria and were subsequently included in the study. The selection process, which is summarized in Figure 1, resulted in a total of 22 articles. The metadata from this final selection can be found in Table 1. All of the included studies were published from 2002 onwards, although no data restriction was applied.

### Overview of the analysed articles

The selected articles reported on studies that investigated a broad range of topics in different settings. Their designs and methodology varied: nine were qualitative, 10 were quantitative and three used mixed methods [27, 33, 38]. As expected, compared to the quantitative studies, the qualitative studies presented in-depth data from a relatively small number of cases (range 7–98). In contrast, the quantitative studies made use of predetermined variables and included more cases (range 461–85,760). All of the qualitative studies made use of semi-structured interviews and/or focus groups while all of the quantitative studies either conducted and/or analysed (telephone) survey data. Most of the studies included patients with an average age well above 50 years, and included both men and women.

Most studies focused specifically on patients with multiple conditions (73%). In all of the publications, multimorbidity (or comorbidity) was defined as having at least two related or unrelated conditions. Both primary and secondary care settings were represented. All of the included studies were undertaken in high-income countries.

### Synthesis of the experiences of patients with multimorbidity

**Figure 2:**
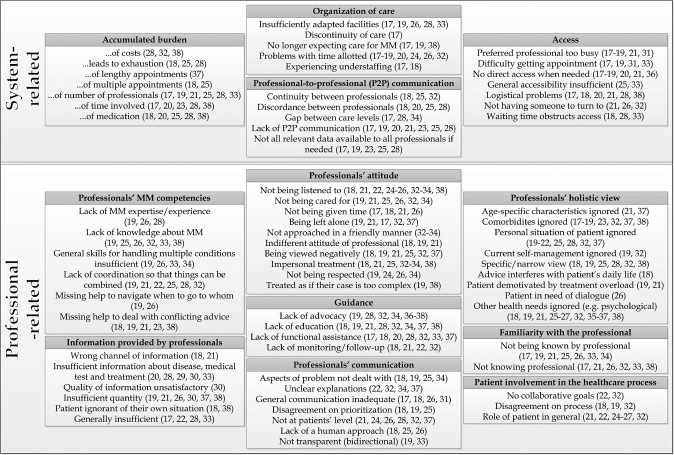
Self-reported experiences of patients with multimorbidity (MM) with their healthcare process (synthesizing qualitative and quantitative evidence).

The selected studies contained 540 statements describing self-reported experiences of patients with multimorbidity, relating to both quantitative and qualitative evidence. Thematic analysis of both types of evidence revealed 12 categories of experiences with the healthcare process (Figure 2). A lack of a holistic view by a professional was mentioned most often (73% of the articles). Other important experienced problems with the healthcare process were related to professionals’ communication (64%), professionals’ attitude and their information provision (both 59%). Patients also mentioned system-related problems, of which the difficulty to access the healthcare system was most important (59%). The self-reported experiences of patients with multimorbidity were thus rooted in both system- and professional-related aspects. However, professional-related experiences were mentioned most often, and also more diverse based on a subdivision into eight categories (compared to four system-related categories). The following sections describe the experiences of patients with multimorbidity for each category based on evidence in both the qualitative and quantitative studies included, as summarized in Figure 2.

### System-related experiences

There were four categories of patient experiences related to the healthcare system: ‘access’, ‘accumulated burden’, ‘organization of care’, and ‘professional-to-professional communication (P2P communication)’. Access was considered problematic because it was not clear to patients with multimorbidity where they should go and whom they should see, and because of the inaccessibility of preferred professional and care when this was needed promptly. These experiences were reflected in difficulties that were mentioned and problems when making appointments. Accumulated burden referred to experiences with regard to a substantial increase in the amount of care due to having multiple conditions, (e.g. time, cost, or polypharmacy). Patients relate these experiences to the system, which poses the problem of not being able to respond to patients in an integrated way. Experiences concerning the organization of care related to discontinuity of the process, insufficient time allotted, understaffing, and facilities that are not sufficiently adapted to their needs. P2P communication referred mainly to an experienced informational discontinuity in the communication between professional.

Although P2P communication might be considered to be professional-related, patients considered this a system issue. Source data pointed to acceptance strategies among patients, attributing their negative care experiences to system failures (e.g. for hurried and impersonal interactions due to insufficient time). Sometimes patients no longer even expected proper care: “There’s no way they could ever keep up with all of the things that you’ve got” [19].

### Professional-related experiences

Professional-related aspects included both the conduct of individual professionals and the perception of all professionals as a whole. The term ‘professionals’ referred to both doctors and other medical staff. Professional-related experiences comprised eight categories: ‘information provision by professionals’, ‘patient involvement in the healthcare process’, ‘guidance’, ‘familiarity with the professional’, ‘professionals’ attitude’, ‘professionals’ communication’, ‘professionals’ multimorbidity competencies’, and ‘professionals’ holistic view’. Information provision was experienced as problematic in the adaptation to the most suitable channel of information and in the level of information provided. Insufficient patient involvement was reflected in disagreement about the healthcare process and not determining goals collaboratively.

Patients experienced a lack of guidance in navigating the system, which was experienced as the lack of an advocate in their healthcare process. They feel unqualified to manage their multiple difficulties alone and desire a greater degree of assistance [17]. The Australian Chronic Disease Management Office indicated that such a guide empowers patients; for example, through education and functional assistance regarding self-management [28]. Patients experienced familiar professionals to be best placed to assess their situation and abilities. Patients with multimorbidity considered discontinuity among professionals to be problematic because of the challenges of building a therapeutic relationship.

The professionals’ attitude was mentioned in two thirds of the studies, and referred to the patients’ perception of insufficient listening, care, and respect. Feeling like “an island of illness” [19] illustrates how patients reported on experiencing their lonely “battle” [21]. Professional communication style was a theme in many studies. Patients found explanations provided by professionals to be unclear, exceed their level of understanding, and lack human interaction. They felt that professionals’ multimorbidity competencies were generally insufficient, reflected by a lack of specific multimorbidity experience, knowledge, and coordination skills among professionals. Patients reported a need for a system or professional who is able “to keep track of multiple shifting priorities” [21]. The patients connected most experiences to a lack of a holistic view of their illness by the professionals. Patients expressed that holistic care would consider their age, social context, comorbidities, and daily life. Frequently, the lack of a holistic view was indicated to stem from an approach that was (too) restricted to a single condition. The absence of a holistic view also meant that multiple medical as well as psychological needs of patients went unmet.

### Interrelatedness of experiences

**Figure 3:**
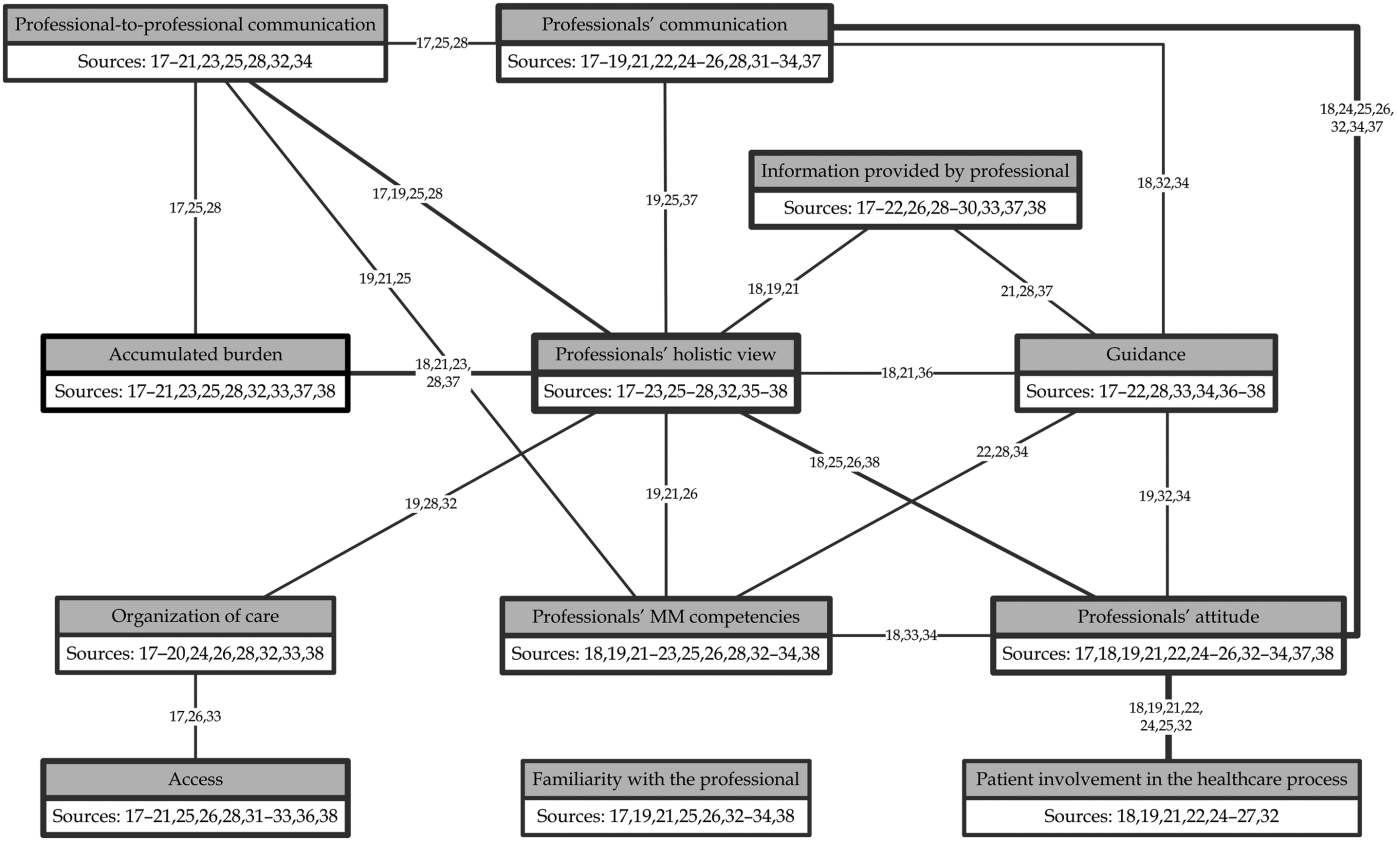
Overview of self-reported experiences of patients with multimorbidity (MM) and their interrelatedness (when mentioned by three or more sources). Source numbers correspond to literature provided in the reference list of this article. Weight of the lines framing the text boxes (categories) indicates the number of sources mentioning that experience. Weight of the connecting lines indicates the number of sources that mentioned the link.

Many relationships between categories of experiences were identified based upon patients’ self-reported interrelatedness between different aspects of care (Figure 3). The number of sources that mentioned each connection provided an indication of interrelatedness of experiences of patients with multimorbidity. It is important to note that the methodology does not allow the figure to be interpreted quantitatively. Analysis shows that professionals’ lack of a holistic view was not only an important experience to patients with multimorbidity but also related to many other experiences (8 associated categories). This is indicated by the central position of ‘holistic view’ in Figure 3 (8 connecting lines). Professionals’ attitude (5), insufficient guidance in navigating the healthcare process (5), and a lack of multimorbidity competencies among professionals (4) were also dominant in the experiences of patients with multimorbidity. Furthermore, the professionals’ attitude is prominent due to the robust connection with professionals’ communication and patient involvement. Overall, synthesizing the evidence resulted in a complex web of interrelated experiences, to which the qualitative studies contributed mostly.

## Discussion

This scoping review synthesized evidence on self-reported experiences of patients with multimorbidity into 12 categories. These included both system- and professional-related experiences. Professionals failing to adopt a holistic approach with their patients seemed to be the overarching experience - mentioned most often and being interrelated to most other experiences. In general, professional-related issues were dominant in the experiences of patients with multimorbidity. This is consistent with previous studies that reported the pivotal role of the patient-professional relationship in determining the patient experience of their care [e.g. 39]. However, our scoping review showed that patients were also sensitive to system-related issues.

### Strengths and weaknesses

A review is limited by the existing body of evidence. The limited number of studies relating to the experiences of patients with multimorbidity, and the recency of their publication - the oldest study was published in 2002 - illustrates the novelty of this field of study. This study depends upon the methodology, design and data that have been published in those source articles. Because most of these sources did not report on the positive experiences, this study rather provides an overview of the problems of patients with multimorbidity.

The choice of methodology, a scoping review, may also have contributed to not finding many positive experiences. A scoping review may even miss important studies since it does not aim to assess all literature systematically. For instance, we used the search terms “multimorbidity” and “comorbidity”, while “concurrent conditions”, for example, may have yielded additional studies. Moreover, we did not explore sources cited by the literature that came up in the search. However, a scoping review does not require identification of all available studies. Furthermore, the quality of the source studies was not formally assessed in this scoping review – as the methodology does not require it – but it is a limitation that may affect the results. However, all articles were published by peer-reviewed journals and, as such, are expected to be of a good standard. Nevertheless, with this limitation in mind, it is important not to interpret the overview (Figure 3) quantitatively, because numbers depend on the selected studies. In other words, a scoping review cannot state *how much* experiences are interrelated. Nevertheless, this type of review enables us to obtain insight into *how* experiences of patients with multimorbidity interrelate, with the data being interpreted in a qualitative way.

Thematic analysis was used to identify overarching themes, which draws together and gives perspective to the existing discrete studies. Thematic analysis does not assign weights in synthesizing different types of data. However, this was not required in order to gain a broad overview of the experiences of patients with multimorbidity. The significance of this study lies in providing that coherent understanding by synthesizing different types of evidence. This overview could provide a basis for hypothesis generation with regard to how healthcare could be adjusted to respond specifically to the needs of patients with multimorbidity. Future research must advance our knowledge of this important and growing group of patients.

Furthermore, we approached multimorbidity as having two or more conditions concomitantly. However, this generic concept disregards the enormous diversity among cases of multimorbidity, varying widely in severity of illness, types of illness, numbers of illnesses and contextual factors. Future research should consider these variations and further explore patient experiences relating to various combinations of factors. Regardless of the varied presentations of patients with multimorbidity, this study shows having multiple conditions leads to similar experienced problems.

### Meaning of the study

The results of this review provide a coherent understanding of the phenomenon of multimorbidity and cover both system- and professional-related aspects. Synthesized patient experiences point at the dysfunctionality of medical professionals being organized in a disease-by-disease approach when working with patients with multimorbidity [40]. Interrelatedness of experiences (Figure 3) reveals the lack of holistic care as the overarching theme that patients with multimorbidity encounter. Therefore, the results question the dominance of the ‘single-disease/single medical specialist’ model upon which healthcare delivery is currently based [41]. The results call for a greater emphasis on the generalist skills of healthcare professionals [42, 43].

Patient experiences are critical inputs for improving multimorbidity care, and this is supported by this scoping review. Current efforts to adapt care to multimorbidity seem to focus on increasing the *operational excellence* of healthcare (e.g. through concepts like integrated care and self-management). Patient experiences are mostly not considered in these efforts to improve organizational structures. This review indeed confirms that patients mainly report on professional-related aspects. However, patients notice both professional conduct and system-related aspects of the healthcare process, and consider them to be interrelated. Thus, patients are also aware of structural and organizational aspects of their healthcare provision and even connect them to their experiences of interacting with their healthcare providers. These results indicate that improving the healthcare process requires adjustment of both system- and professional-related aspects and consideration of their interrelatedness.

The review also contributes to the understanding of the experiences of patients with multimorbidity compared to patients with single conditions, although no comparison is made. Most experiences reported by patients with multimorbidity are also found in literature on patient experiences in general [e.g. 44]. In addition, Hewitson *et al*. [45] show that experiences of patients with multimorbidity are not so much different from patients suffering from a single condition, but are rather more pronounced for them (e.g. time slot). Experiences could, therefore, be understood as a continuum, in which multimorbidity influences the position on this continuum negatively. For example, discordance in priorities may be experienced with a single professional [46] and become more likely when more professionals are involved.

## Conclusions

This scoping review adds to the evidence base underlying healthcare delivery for patients suffering from multiple conditions by analysing qualitative and quantitative empirical evidence on their experiences. To date, a coherent understanding of the perspective of patients with multimorbidity did not exist. Although multimorbidity is not a generic concept – there are infinite combinations of conditions and contextual factors – this study shows that having multiple conditions in general leads to similar problems in the healthcare process. These regard both the conduct of professionals and organizational aspects. Although the methodology of a scoping review limits the studies included, a coherent overview of patient experiences was gained by synthesizing different types of data and revealing overarching themes; of which the lack in a holistic view of professionals seems most important.

Building upon these results, specific efforts could address the challenges of multimorbidity more comprehensively, rather than focusing on single aspects of the healthcare process. For instance, clarification of the person responsible for the patient with multimorbidity when several professionals are involved should take place but also must be communicated effectively to the patient. The understanding of patients’ experiences and healthcare needs of patients with multimorbidity gained from this analysis can contribute to improving care for this important patient group.

## Figures and Tables

**Table 1:** Characteristics of the studies included in the scoping review (*n*=22).

Study/Reference	Multimorbidity focus of study	Study design	N	Geographical setting	Institutional setting	Study population	
						Age (mean±SD)^*^, years	Male sex, %
Adeniji *et al.*, 2015 [20]	Primary	Quantitative: survey	486	UK	Primary care	31–91 (70±10)	48
Bayliss *et al.*, 2008 [21]	Primary	Qualitative: semi-structured interviews	26	USA	Health maintenance organization	65–84	50
Boult *et al.*, 2008 [22]	Primary	Quantitative: analysis of telephone survey data	904	USA	Primary care	66–96 (78.1)	44.6
Burgers *et al.*, 2010 [23]	Primary	Quantitative: analysis of telephone survey data	8,973	Australia, Canada, France, Germany, The Netherlands, New Zealand, UK, USA	Primary and secondary care	18–≥65	36
Cowie *et al.*, 2009 [17]	Primary	Qualitative: semi-structured interviews	33	UK	Primary care	42–83	52
Fung *et al.*, 2008 [24]	Primary	Quantitative: analysis of telephone survey data	15,709	USA	Not specified	NS (45.8±17.1)	52.2
Gallagher *et al.*, 2013 [25]	Secondary	Qualitative: semi-structured interviews	33	Ireland	Primary care	NS–84 (44.5)	38
Grundberg *et al.*, 2014 [26]	Primary	Qualitative: semi-structured interviews	7	Sweden	Mental health promotion	83–96	14
Kjeken *et al.*, 2006 [27]	Secondary	Quantitative and qualitative: survey	1,193	Norway	Not specified	NS (59.6±15.6)	26
Maneze *et al.*, 2012 [28]	Primary	Qualitative: semi-structured interviews	13	Australia	Emergency care	37–80	62
Nicolaije *et al.*, 2012 [29]	Secondary	Quantitative: survey	742	The Netherlands	Cancer care	NS (66.7±8.5)	0
Noël *et al.*, 2005 [18]	Primary	Qualitative: focus groups	60	USA	Primary care	30–80	80
Oerlemans *et al.*, 2012 [30]	Secondary	Quantitative: survey	1,135	The Netherlands	Cancer care	NS (61.6)	59.6
Paddison *et al.*, 2015 [31]	Secondary	Quantitative: survey	85,760	UK	Diabetes care	18–≥85	41
Penn *et al.*, 2002 [32]	Primary	Qualitative: focus group	7	USA	Mental care	22–55	0
Rincón-Gómez *et al.*, 2011 [33]	Primary	Quantitative: survey; Qualitative: interviews	461	Spain	Primary care	NS (74.3±9.6)	75.4
Shadmi *et al.*, 2006 [34]	Primary	Quantitative: survey	120	USA	Primary care	NS (75.4)	66.7
Sperling *et al.*, 2014 [35]	Secondary	Quantitative: survey	4,401	Denmark	Cancer care	18–≥70	48
Urbanoski *et al.*, 2008 [36]	Primary	Quantitative: analysis of survey data	4,052	Canada	Mental care	15–≥60	45
Whitson *et al.*, 2011 [37]	Primary	Qualitative: semi-structured interviews	98	USA	Outpatient care	NS (80.4±7.8)	33
Williams, 2004 [19]	Primary	Qualitative: semi-structured interviews	12	Australia	Acute care	34–77 (60.9)	50
Williams *et al.*, 2007 [38]	Primary	Quantitative: survey; Qualitative: interviews	20	Australia	Acute care	NS (67.1±6.6)	35

NS, not specified; SD, standard deviation. ^*^Where provided.
